# Supplementary material for: Introducing Hann windows for reducing edge-effects in patch-based image segmentation
Source: PLoS One. 2020 Mar 12;15(3):e0229839. doi: 10.1371/journal.pone.0229839 (PMC7067425; doi:10.1371/journal.pone.0229839)
Supplement: Attachment — Submitted filename: rebuttal_letter_2nd.pdf [file pone.0229839.s002.pdf]

Nicolas Pielawski
Uppsala University
Lägerhyddsvägen 2
752 37, Uppsala
nicolas.pielawski@it.uu.se

February 14th 2020

Dear Editor,

We thank the reviewers for their constructive comments and have revised the manuscript to address their concerns.

The points we addressed are the following:

- The author showed the experiment result of Dice in Fig 4, but didn't give the overall performance comparison in terms of Dice
  We added a new figure (fig 5), containing statistical testing of the different method with respect to the baseline and triangular/cui.
- The discussion is not sufficient.
  The discussion section was modified and some content was added.

The changes are colour coded in the following fashion: blue for changes, green for added text, red for removal and yellow represents the parts that were moved to make a more logical flow adding the information about the Dice evaluation and clarifying the SSIM evaluation.

We hope that the revised manuscript now meets PLOS ONE's publication criteria.

Sincerely yours,

Nicolas Pielawski (corresponding author)
Address: Polacksbacken (Lägerhyddsvägen 2), 753 37 Uppsala, SWEDEN
E-mail: nicolas.pielawski@it.uu.se
