# Supplementary material for: Introducing Hann windows for reducing edge-effects in patch-based image segmentation
Source: PLoS One. 2020 Mar 12;15(3):e0229839. doi: 10.1371/journal.pone.0229839 (PMC7067425; doi:10.1371/journal.pone.0229839)
Supplement: Attachment — Submitted filename: rebuttal_letter.docx [file pone.0229839.s001.docx]

Nicolas Pielawski

Uppsala University

Lägerhyddsvägen 2

752 37, Uppsala

[nicolas.pielawski@it.uu.se](mailto:nicolas.pielawski@it.uu.se)

January 11^th^ 2020

Dear Editor,

We thank the reviewers for their constructive comments and have revised the manuscript to address their concerns.

The points we addressed are the following:

- References: I find it hard to believe that only 11 references were relevant for this manuscript. The authors should significantly improve their literature review, in order to place their work more rigorously within existing literature.

The issue is relatively little discussed as many researchers have access to powerful computers or can reformulate the problem to deal with smaller images. One approach consists of cropping the borders (e.g. Rudolph, Robert, et al. "Efficient identification, localization and quantification of grapevine inflorescences in unprepared field images using fully convolutional networks." arXiv preprint arXiv:1807.03770 (2018)) but can still generate artifacts.

- Although a mention of the general complexity of their method is shown, it would be very much helpful for the reader to display the computational time for the authors technique – along with the benchmark methods.

Time complexity is defined as <number of patches> times <time complexity of the deep learning model> and thus it will vary from model to model, computer to computer and so on. Using overlapping windows increases the number of patches by a factor of 4 and increases the complete time complexity by the same factor.

- The SSIM should be presented in detail and not just put in a reference other established accuracy metrics (such as overall accuracy, precision, recall and F1 score) be also fruitful?

Agreed. We added a new metric: the DICE coefficient, which corresponds to the F1 score.

- Eq 6 to Eq 10 are not clear enough, especially the definition of w(i).

The text was modified to clarify and provide a better explanation of the equations.

- It is necessary to add commonly used metrics for segmentation tasks, such as DICE.

Agreed, we added two more experiments (see fig. 3).

- Eq 10, should be if i <= L/2 and j <= J/2.

Corrected.

- Was a different color look-up table used for the pyramidal window? […] Was it necessary to compensate for this different integrated area by using a different color look-up table, or alternatively, were the data normalized prior to visualizing as a 2D heatmap?

The same colour look-up tables were used in the article. The un-normalizing windows were normalized, it wasn’t specified in the original article, corrected now.

- The description of the different approaches and different methods is a bit confusing on page 5, for the top 3 paragraphs. For example, the first paragraph refers to "The five different approaches", while the 3rd paragraph (which is the Fig. 3 caption) refers to "the adjusted SSIM for six different methods". It is a bit unclear of what an "approach" is vs. a "method" and whether there are 5 or 6 of them.

Changed all occurrences of “approach” to “method”, and 5 to 6. There are 5 methods plus 1 baseline, clarified in the revised article.

- In Fig. 3a, there appear to be 5 different window functions shown. For similar reasons, the 2nd paragraph on page 5 is also confusing, in the sentence that reads "Due to a low variance in the SSIM indices for each method but a high variance between methods..." It is unclear here as well what the method is that the sentence is referring to. Clearing up the language in these 3 paragraphs is needed. The authors might help to clarify the vertical axis of Figure 3 by explaining that this is the improvement in SSIM over baseline.

The three paragraphs were partially reformulated.

- On page 6, first paragraph of the Discussion section, the 2nd sentence contains the word "theory" twice.

Corrected.

The changes are colour coded in the following fashion: blue for changes, green for added text, red for removal and yellow represents the parts that were moved to make a more logical flow adding the information about the Dice evaluation and clarifying the SSIM evaluation.

We hope that the revised manuscript now meets PLOS ONE’s publication criteria.

Sincerely yours,

Nicolas Pielawski (corresponding author)

Address: Polacksbacken (Lägerhyddsvägen 2), 753 37 Uppsala, SWEDEN

E-mail: [nicolas.pielawski@it.uu.se](mailto:nicolas.pielawski@it.uu.se)
